# Irisin Pretreatment Protects Kidneys against Acute Kidney Injury Induced by Ischemia/Reperfusion via Upregulating the Expression of Uncoupling Protein 2

**DOI:** 10.1155/2020/6537371

**Published:** 2020-08-31

**Authors:** Rui Zhang, Jing Ji, Xiaoshuang Zhou, Rongshan Li

**Affiliations:** ^1^Shanxi Medical University, Shanxi, China; ^2^Department of Nephrology, The Affiliated People's Hospital of Shanxi Medical University, Shanxi Provincial People's Hospital, Shanxi Kidney Disease Institute, Taiyuan, Shanxi 030001, China

## Abstract

As a common disorder, acute kidney injury (AKI) is characterized by high mortality and morbidity, and current therapeutic options for AKI remain limited. Irisin, a muscle factor, plays an important role in metabolic disorders. However, the role of irisin in AKI is still unclear. To assess the effect of irisin on the course of AKI, we used an ischemia/reperfusion (I/R) C57BL/6 mouse model. Supplementation with irisin attenuated kidney injury induced by I/R, as shown by decreases in the levels of serum creatinine and blood urea nitrogen. Animal model studies also showed that irisin pretreatment upregulates the expression of uncoupling protein 2 (UCP2) and protects against the renal cell apoptosis and oxidative stress caused by I/R. *In vitro*, hypoxia/recovery (H/R) treatment was applied to induce tubular cell apoptosis. Irisin pretreatment ameliorated the cell apoptosis induced by H/R, while transfection of UCP2 siRNA significantly reduced the protective effect of irisin in cells after H/R. In addition, AMPK signaling may be involved in irisin-mediated upregulation of UCP2 in a renal proximal tubular epithelial cell (PTEC) model. Thus, the renoprotective effect of irisin on AKI may be mediated through increasing the expression of UCP2 in kidneys after I/R.

## 1. Introduction

Acute kidney injury (AKI) is a worldwide public health problem characterized by a short-term increase in serum creatinine (SCr) and blood urea nitrogen (BUN) [1]. The incidence of AKI is 21.6% among adults and 33.7% among children, and AKI also affects 1 in 5 hospitalized patients [2]. Yang et al. [3] reported that there are 1.4 to 2.9 million patients with AKI in China and that total expenses due to AKI are approximately 13 billion dollars. Although preventative strategies and supportive measures for AKI have improved, the morbidity and mortality of AKI are still high [4]. Although many factors, such as ischemia, nephrotoxic agents, and sepsis, can cause AKI, ischemia-reperfusion (I/R) is the most frequent causative factor of AKI.

Uncoupling proteins (UCPs) are mitochondrial inner membrane proteins. There are five mammalian UCPs, UCP1–5, among which uncoupling protein 2 (UCP2) is widely found in the kidney tissue [5]. Numerous recent studies [6, 7] have indicated that UCP2 protects against I/R-induced AKI. Furthermore, through interaction with UCP2, irisin, a hormone secreted by skeletal muscles immediately after exercise and encoded by the fibronectin type III domain-containing 5 (FDNC5) gene, has a protective effect on lung damage caused by ischemia-reperfusion (I/R) [8]. However, whether irisin has certain effects on AKI remains unknown. Above all, we proposed that irisin pretreatment played a considerable role in the course of AKI by regulating the expression of UCP2. In this study, a I/R mouse model and cell apoptosis in a PTEC model induced by hypoxia/reoxygenation (H/R) were used to verify this hypothesis. We found that irisin pretreatment has a renoprotective effect against I/R injury in mice and *in vitro*. Furthermore, the renoprotective effect of irisin on AKI was attenuated by transfection of UCP2 siRNA *in vitro*.

## 2. Materials and Methods

### 2.1. Animal Model

Specific pathogen-free male C57BL/6 mice, 6-8 weeks old, were purchased from the Animal Center of Shanxi Medical University (Taiyuan, China). All mice were housed with 12 h light/dark cycles. All programs and experiments were performed based on protocols from the Affiliated People's Hospital of Shanxi Medical University Institutional Animal Care and Use Committee. To increase UCP2, the mice were fed a standard semipurified diet with or without 1% conjugated linoleic acid (CLA). The materials of the diet were the same as those described in previous studies [6, 7].

Furthermore, to investigate the role of irisin in kidney I/R injury, the mice were randomly allocated into four groups: sham group, sham+irisin group, I/R group, and I/R+irisin group. In the irisin-treated groups, the mice were given an intraperitoneal injection of different irisin doses (Cayman Chemical, MI, USA) (10, 100, and 200 *μ*g/kg/day) for 14 days before I/R surgery. An equal volume of saline was administered to the mice in the control and I/R groups.

Mice underwent renal I/R surgery according to an established procedure [9, 10]. Briefly, all animals were anesthetized with 1% pentobarbital sodium (5 mg/100 g body weight), and an abdominal midline incision was made. Then, microaneurysm clamps were used to clip the bilateral renal pedicle. After 45 min, the clamps were removed for renal reperfusion. The mice in the sham and sham+irisin groups underwent a sham operation that consisted of the same procedure except clamping of the bilateral renal pedicle. After 24 h, blood and kidney tissues were obtained for further experiments.

### 2.2. Renal Proximal Tubular Epithelial Cell (PTEC) Culture and Treatments

The HK-2 cells were purchased from Wuhan Hualian Biotechnology. The cells were cultured in Dulbecco's modified Eagle's medium (DMEM)/F12 (Gibco, USA) supplemented with 5% fetal bovine serum (FBS) and 1% penicillin streptomycin at 37°C in an atmosphere with 5% CO_2_. When cells grew to 80% confluency, a hypoxia/recovery (H/R) model was constructed. In brief, the cells underwent hypoxia treatment for 3 h (5% CO_2_, 1% O_2_, and 94% N_2_) and then received reoxygenation for 3 h (normal culture conditions). Furthermore, before H/R, cells were treated with recombinant irisin (1 *μ*g ml^−1^, Cayman Chemical, MI, USA). In some experiments, cells were pretreated with compound C (CC, 5 *μ*M, Sigma-Aldrich) or vehicle before adding irisin.

The siRNA transfection reagent (GE Healthcare Dharmacon, USA) was used to transfect UCP2 siRNA or nontargeting siRNA (GenePharma, Shanghai, China) into cells. UCP2 plasmid was purchased from Hanbio Biotechnology, and LipoFiter 3.0 (Hanbio Biotechnology, Shanghai, China) was used for UCP2 or cDNA3 plasmid (p-UCP2, p-cDNA3) transfections.

### 2.3. Evaluation of Renal Function

To evaluate renal function, BUN and SCr were assessed. After coagulation, blood samples were centrifuged to obtain serum. BUN was quantified with a colorimetric assay kit, and SCr was investigated by a creatinine assay kit (Nanjing Jiancheng, Nanjing, China).

### 2.4. Histology Assays

Kidney tissues were fixed with 10% formalin, embedded in paraffin, and sectioned at 3 *μ*m for hematoxylin and eosin staining. Renal injury was evaluated by experienced renal pathologists in a blinded manner. According to a previous study [11], tubular injury scores were assessed, and the scores ranged from 0 to 4 as follows: 0, less than 10% of kidney tissue damaged; 1, 10%-25% of kidney tissue damage; 2, 25%-50% kidney tissue damage; 3, 50%-75% kidney tissue damage; and 4, more than 75% kidney tissue damage. At least ten fields were selected at random, and an average score was calculated.

### 2.5. Immunohistochemical Staining

After deparaffinization in xylene and rehydration through an ethanol series, the sections (3 *μ*m) were washed in PBS buffer and incubated with 3% methanolic hydrogen peroxide and 10% standard goat serum. Following antigen retrieval, the slides were incubated with primary antibodies against intercellular adhesion molecule- (ICAM-) 1 (1 : 2000, Abcam, CA, USA) and P-selectin (1 : 100, Boster, Wuhan, China) at 4°C overnight. After washing with PBS, the slides were incubated in horseradish peroxidase-conjugated goat anti-rabbit secondary antibody (Boster, Wuhan, China) for 30 min at 37°C. After visualization with 3,3-diaminobenzidine tetrahydrochloride (Boster, Wuhan, China), hematoxylin was used to counterstain the sections. Finally, the images were obtained using Nikon light microscopy (Nikon Ni-U, Japan). All images were evaluated with ImageJ software (NIH, Bethesda, MD, USA).

### 2.6. Western Blotting (WB)

The HK-2 cells and kidney tissues were extracted with a Radio Immunoprecipitation Assay (RIPA) lysis buffer with PMSF (10 mM, Boster, Wuhan, China) and a phosphatase inhibitor cocktail (10 mM, Boster, Wuhan, China) on ice. The concentration of protein was determined using a BCA Protein Assay Kit (Boster, Wuhan, China). The same amounts of protein samples were separated by electrophoresis and transferred to a polyvinylidene difluoride membrane (Millipore, MA, USA). After blocking with the blocking buffer at 37°C for 2 h, the membranes were then incubated with primary antibodies overnight at 4°C: UCP2 (1 : 1000, Abcam, CA, USA), cleaved caspase 3 (1 : 1000, Cell Signaling Technology, MA, USA), interleukin- (IL-) 1*β* and 6 (IL-6) (1 : 500, Santa Cruz, USA), kidney injury molecule- (KIM-) 1 (1 : 500, Boster, Wuhan, China), AMP-activated protein kinase (AMPK) and p-AMPK (1 : 1000, Cell Signaling Technology, USA), and *β*-actin (1 : 10000, Abcam, CA, USA) (used as an internal reference). Next, the membranes were incubated at room temperature with corresponding peroxidase-conjugated IgG antibody (1 : 5000) for 1 h. Finally, the membranes were imaged by ECL and analyzed with the ChemiDoc MP (Bio-Rad, CA, USA).

### 2.7. Terminal Deoxynucleotidyl Transferase-Mediated dUTP Nick-End Labeling (TUNEL) Detection

The paraffinized kidney sections were prepared according to the instructions of a TUNEL Apoptosis Assay Kit (Nanjing KeyGen Biotech, Jiangsu, China), and nuclei were stained with DAPI (Boster, Wuhan, China). For the relative quantification of apoptotic cells, 10 random regions per section were examined with an upright fluorescence microscope (Nikon Ni-U, Japan), and the number of positively stained cells was counted. At least three sections were selected for each group.

### 2.8. Real-Time Reverse Transcription-Polymerase Chain Reaction Analysis

According to the manufacturer's protocols, the TaKaRa extraction kit was used to extract the total RNA from kidney tissues, and cDNA was immediately synthesized (TaKaRa Bio, Tokyo, Japan). Real-time quantification was then performed by using a SYBR Green PCR Kit (TaKaRa Bio, Tokyo, Japan) and a Bio-Rad CFX96 Fast real-time PCR system. To analyze mRNA expression data, the 2^-*ΔΔ*CT^ method was used, and expression of target genes in the kidney was normalized to *β*-actin. The primers used for RT-RCR are listed in Table 1.

### 2.9. Flow Cytometry Analysis

An Annexin V-FITC/PI detection kit (Nanjing KeyGen Biotech, Jiangsu, China) was performed to detect cell apoptosis. HK-2 cells were cultured in 6-well plates and then incubated overnight and divided into different groups as follows: (1) control group (CTL); (2) transfected with or without irisin; (3) transfected with p-UCP2; and (4) treated with CC with or without irisin. The cells then underwent hypoxia/reoxygenation treatment. After digestion with trypsin without EDTA, cells were harvested, stained with Annexin V-FITC and PI, and detected by a Navios flow cytometer (Cytomics FC 500, USA). The cell apoptotic ratio was determined as the sum of early and late cell apoptotic ratios.

### 2.10. Tumor Necrosis Factor- (TNF-) *α*, Irisin, and Oxidative Stress

The levels of TNF-*α* and irisin were measured using sandwich ELISA kits based on the manufacturer's protocols (Elabscience, Wuhan, China; Bioswamp Life Science, Wuhan, China). After kidney tissues were lysed in phosphate-buffered saline (PBS), the levels of myeloperoxidase (MPO), malondialdehyde (MDA), superoxide dismutase (SOD) were measured according to the manufacturer's instructions (Nanjing Jiancheng, Nanjing, China).

### 2.11. Statistical Analysis

All the values are presented as mean ± SEM. Statistical significance between the data obtained from two independent groups was assessed by Student-Newman-Kuel's unpaired tests, which were conducted using data obtained from more than 3 groups by one-way ANOVA, followed by Tukey's test. The value of *P* < 0.05 indicated statistical significance. All the statistical tests were conducted by GraphPad Prism Software version 7.0 (GraphPad Software, CA, USA).

## 3. Results

### 3.1. Irisin Pretreatment Alleviates Kidney I/R Injury

**Figure 1:**
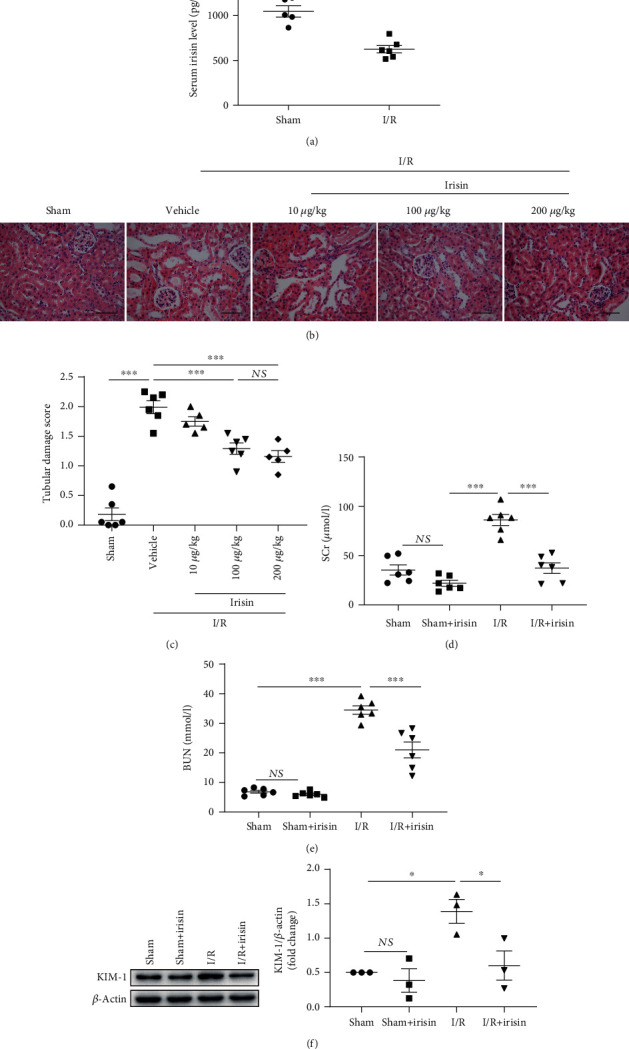
I/R injury caused a reduction of the irisin level in serum, and irisin pretreatment ameliorated renal injury after ischemia/reperfusion injury in the mouse model. (a) Ischemia/reperfusion (I/R) reduced the concentration of irisin in mouse serum. (b) Representative images of H&E. Magnification, 400x. Bar = 50 *μ*m. (c) Quantitative analysis of kidney sample tubular injury scores following staining with H&E. Renal function was evaluated by measuring the (d) SCr and (e) BUN levels. (f) Expression of KIM-1 in kidneys detected by Western blotting and quantitative analysis of KIM-1 levels adjusted to those of *β*-actin (*n* = 3 experiments or 6 mice in each group). Data are displayed as mean ± SEM. ^∗^*P* < 0.05; ^∗∗^*P* < 0.01; ^∗∗∗^*P* < 0.001. Abbreviations: NS: no significant difference.

First, we investigated the serum concentration of irisin and found that, compared with sham-treated mice, irisin was significantly decreased in mice subjected to I/R injury (Figure 1(a)). To determine whether irisin supplementation can ameliorate AKI injury induced by I/R, mice were first administered an intraperitoneal injection of irisin of different doses (10, 100, and 200 *μ*g/kg/day). As shown in Figures 1(b) and 1(c), we examined the kidney injury in the I/R mice in each group. Compared with the vehicle-treated group, administration of 200 and 100 *μ*g/kg irisin significantly improved kidney injury and decreased the injury score. However, 10 *μ*g/kg irisin only slightly reduced the kidney injury. And 100 *μ*g/kg irisin was used in the next experiment. BUN and SCr levels and renal morphology were subsequently assessed. As depicted in Figures 1(d) and 1(e), SCr and BUN were significantly increased in C57BL/6 mice after renal I/R. Notably, kidney dysfunction in I/R mice was remarkably alleviated after the administration of irisin, which was demonstrated by a significant decrease in SCr and BUN (Figures 1(d) and 1(e)).

WB was used to detect the expression of KIM-1, which can predict AKI. Compared with the sham group, an increased KIM-1 level was observed in the I/R group; however, after irisin supplementation, the expression of KIM-1 was significantly decreased in I/R mice (Figure 1(f)). In parallel, in the I/R+irisin group, we observed a lower tubular damage score and less renal histopathological damage, including loss of the brush border, dilation of tubules, apoptosis, and shedding of PTECs, compared with the I/R group (Figure 1). The above results indicated that irisin can alleviate renal damage during renal I/R injury.

### 3.2. Irisin Pretreatment Inhibits Cellular Inflammation and Oxidative Stress in Mouse Renal Tissue during I/R Injury

**Figure 2:**
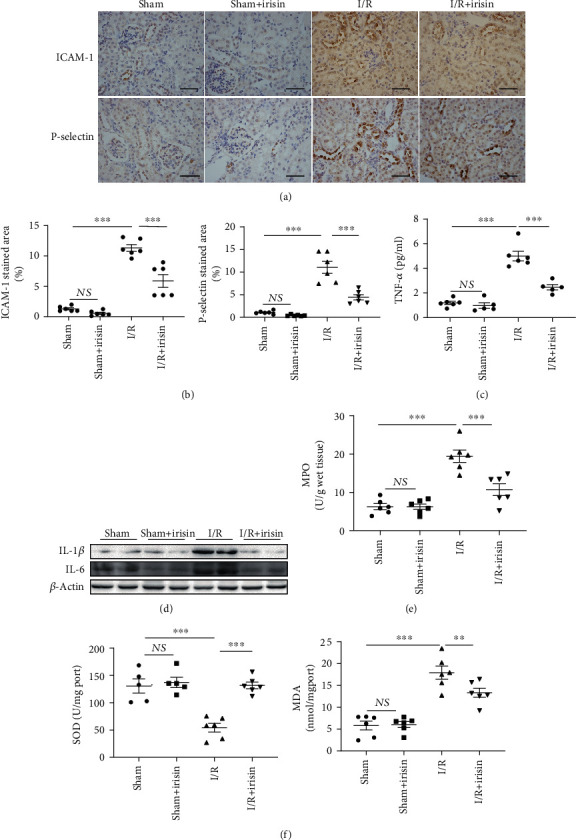
Irisin suppresses the cellular inflammatory response and facilitates oxidative stress in mice after I/R injury. Representative photographs from (a) immunohistochemical and (b) semiquantitative analyses of ICAM-1 and P-selectin. Magnification, 400x. Bars = 50 *μ*m. (c) The level of TNF-*α* measured by ELISA. (d) Representative images of Western blotting of IL-1*β* and IL-6. (e) MPO and (f) SOD and MDA production in the kidney (*n* = 3 experiments). Data are presented as mean ± SEM. ^∗^*P* < 0.05; ^∗∗^*P* < 0.01; ^∗∗∗^*P* < 0.001. Abbreviations: NS: no significant difference.

The cellular inflammatory response and oxidative stress apoptosis play vital roles in AKI [12–14]. To determine whether irisin can reduce renal I/R damage by inhibiting the cellular inflammatory response and oxidative stress, we examined the levels of TNF-*α*, IL-1*β*, IL-6, MPO, ICAM-1, and P-selectin. The immunohistochemical staining showed that the expression of ICAM-1 and P-selectin was significantly decreased by irisin after I/R injury (Figures 2(a) and 2(b)). Additionally, as expected, the levels of inflammatory factors (TNF-*α*, IL-1*β*, and IL-6) were notably suppressed by irisin supplementation before I/R (Figures 2(c) and 2(d)). Many studies have reported that oxidative stress may promote the development of renal injury caused by I/R. As demonstrated in Figure 2(f), compared with the sham group, the MDA level was markedly increased and the SOD level was decreased in I/R mice. However, irisin pretreatment significantly reduced SOD and increased MDA levels relative to those mice in the I/R group. All the data indicated that irisin has a protective effect on the kidney by reducing the cellular inflammatory response and inhibiting oxidative stress in I/R injury.

### 3.3. Irisin Pretreatment Reduces the Apoptosis in Mouse Renal I/R Injury

**Figure 3:**
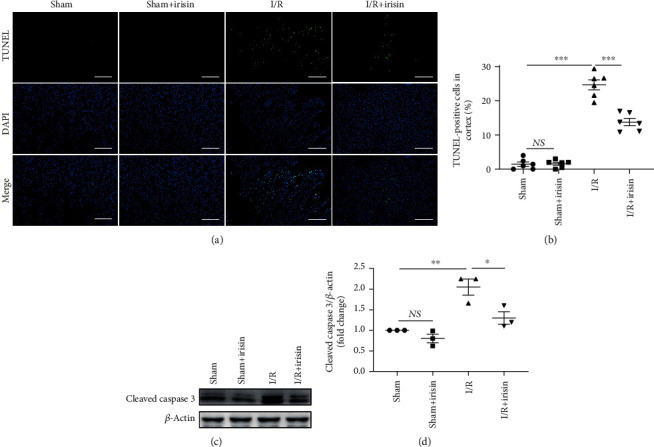
Irisin suppresses I/R-related cell apoptosis in mice. (a, b) Representative images from a TUNEL assay in kidney tissues, and the ratio of TUNEL-stained positive cells in the mouse kidneys from four groups is shown. Magnification, 200x. Bars = 100 *μ*m. (c, d) Representative images of Western blotting and quantitative analysis of cleaved caspase 3 levels normalized to *β*-actin (*n* = 3 experiments). Data are presented as mean ± SEM. ^∗^*P* < 0.05; ^∗∗^*P* < 0.01; ^∗∗∗^*P* < 0.001. Abbreviations: NS: no significant difference.

Tubular cell apoptosis, a vital pathological feature of AKI [15], was assessed by analyzing activation of caspase 3 and TUNEL assay. We observed that irisin pretreatment before I/R markedly reduced the activation of caspase 3 compared with I/R mice (Figures 3(c) and 3(d)). Furthermore, as shown in Figures 3(a) and 3(b), similar results had shown that the ratio of apoptotic cells was decreased in mice that received pretreatment with irisin before I/R injury. Our findings demonstrated that irisin may mitigate renal I/R damage by reducing apoptosis in I/R injury.

### 3.4. Irisin Pretreatment Upregulates UCP2 Expression and Activates AMPK in Murine AKI Induced by I/R

**Figure 4:**
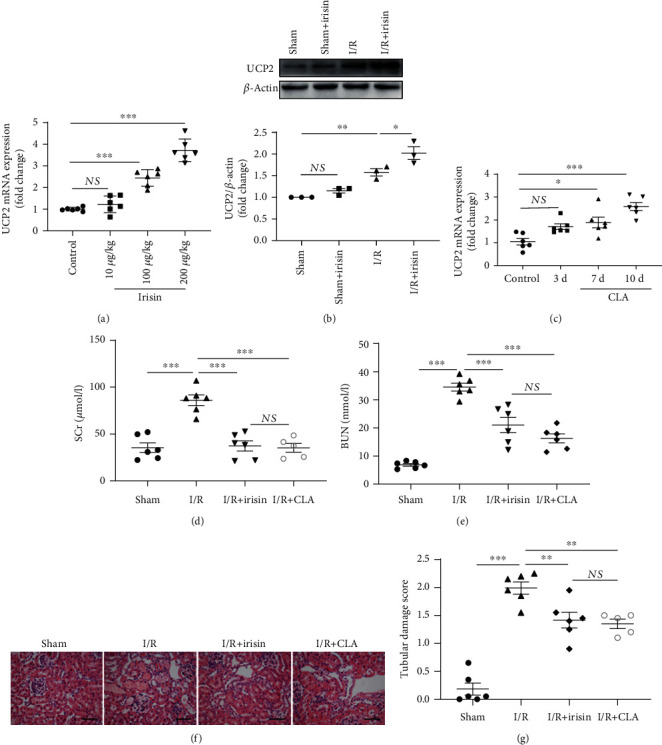
Irisin ameliorates renal I/R damage through regulating UCP2 expression, and UCP2 facilitates I/R-induced renal damage. (a) The mRNA expression of UCP2 in a mouse model subjected to an intraperitoneal injection with different doses of irisin. (b) Western blot illustrating the expression of UCP2 in mice and quantifications of UCP2/*β*-actin. (c) The mRNA expression of UCP2 in a mouse model fed 1% CLA for various durations. Renal function was evaluated by measuring the (d) SCr and (e) BUN levels. (f, g) Representative H&E images and quantitative analysis of kidney sample tubular injury scores following staining with H&E. Magnification, 400x. Bar = 50 *μ*m (*n* = 5-6 mice in each group). Statistics are presented as mean ± SEM. ^∗^*P* < 0.05; ^∗∗^*P* < 0.01. Abbreviations: I/R: ischemia/reperfusion; CTL: control, with no addition of irisin; H/R: hypoxia/reoxygenation; UCP2: uncoupling protein 2; CLA: conjugated linoleic acid; NS: no significant difference.

Compared with the I/R group, supplementation with irisin increased the level of UCP2 mRNA (Figure 4(a)), and administration of 100 *μ*g/kg irisin improved 2.4 times higher than the control group. Furthermore, as shown by the immunoblot in Figure 4(b), after the mice underwent ischemia for 45 min followed by reperfusion for 24 h, the expression of UCP2 was substantially higher than that in the control group. To confirm the UCP2 expression in AKI, the mice were fed 1% CLA. The UCP2 mRNA level markedly increased in renal tissue at 10 days compared with that of the control mice (Figure 4(c)). Additionally, fed with UCP2 significantly improved renal dysfunction exhibited as BUN and SCr (Figures 4(d) and 4(e)). At the meantime, CLA supplementation significantly improved renal proximal tubular injury than the I/R group (Figures 4(f) and 4(g)).

### 3.5. Irisin Pretreatment Suppressed Tubular Epithelial Cell Apoptosis Induced by H/R by Regulating the Expression of UCP2 *In Vitro*

**Figure 5:**
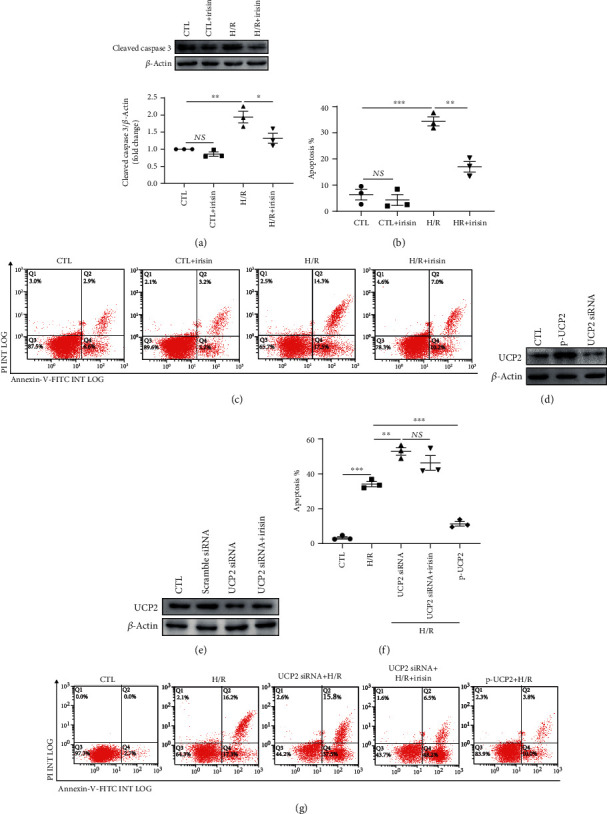
Irisin suppresses cell apoptosis via regulating UCP2. (a) Representative images of Western blotting and quantitative analysis of cleaved caspase 3 levels normalized to *β*-actin *in vitro*. (b, c) The rates of apoptosis in an H/R model in tubular cells (*n* = 3 experiments). (d) Western blot illustrating the expression of UCP2 in cells treated with UCP2 siRNA or p-UCP2. (e) Expression of UCP2 in HK-2 cells pretreated with or without irisin after transfection with UCP2 siRNA or nontargeting siRNA (*n* = 3 experiments). (f, g) The rates of apoptosis in an H/R model in tubular cells transfected with UCP2 siRNA or p-UCP2 with or without pretreatment with irisin versus control groups (*n* = 3 experiments). Statistics are displayed as mean ± SEM. ^∗^*P* < 0.05; ^∗∗^*P* < 0.01. Abbreviations: CTL: control, with no addition of irisin; H/R: hypoxia/reoxygenation; NS: no significant difference.

The functional role of irisin in cell apoptosis and expression of UCP2 was further studied in an H/R cell model. In cultured cells, H/R-induced cleavage of caspase 3 was facilitated in the cells pretreated with irisin (Figure 5(a)). Furthermore, as shown in Figures 5(b) and 5(c), similar results had shown that the ratio of apoptotic cells was decreased *in vitro* that received pretreatment with irisin before H/R injury. As shown in Figure 5(e), after transfected with UCP2 siRNA, the level of UCP2 was significantly decreased in HK-2 cells, and irisin-mediated upregulation of UCP2 was prevented. And the renal proximal tubular epithelial cells were transfected with p-UCP2 to increase UCP2. To clarify the role of UCP2 in H/R injury, we assessed cell apoptosis by flow cytometry to analyze the cell apoptotic ratio. Figures 5(f) and 5(g) show that transfection with p-UCP2 markedly reduced H/R-induced cell apoptosis, and apoptosis in cells transfected with UCP2 siRNA was more pronounced than that in the H/R group. Furthermore, the H/R-induced cell apoptosis was not significantly relieved by irisin in the cells transfected with UCP2 siRNA compared with those transfected with nontargeting siRNA. All these results suggested that the renoprotective effect of irisin on AKI may be mediated by the upregulation of UCP2 in I/R-induced kidney injury.

### 3.6. Irisin-Modulated UCP2 Expression May Be Mediated by Activation of AMPK

**Figure 6:**
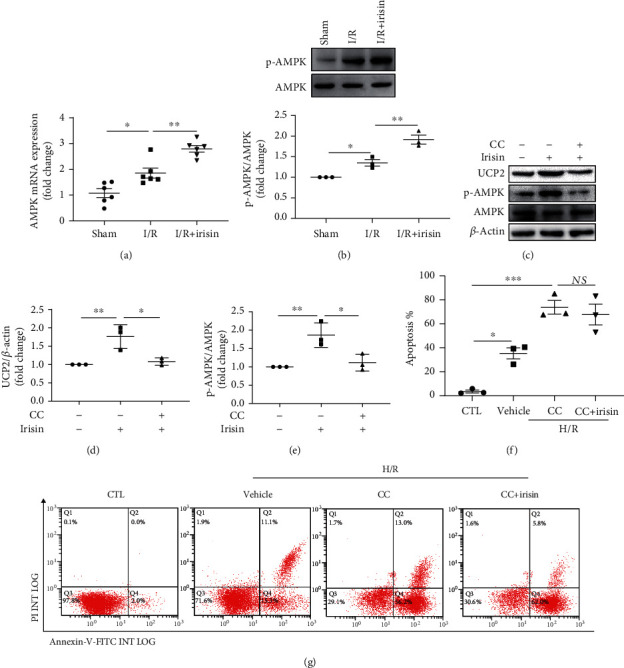
Irisin regulates UCP2 expression in kidneys via the AMPK pathway. (a) The mRNA expression of AMPK in a mouse model. (b) Immunoblot showing p-AMPK levels and the ratio of p-AMPK/AMPK in mice. (c) Immunoblotting shows the UCP2 and p-AMPK levels in an HK-2 cell model treated with irisin with or without CC. Quantitative analysis of the (d, e) UCP2 and p-AMPK levels adjusted to those of *β*-actin and AMPK, respectively (*n* = 3 experiments). (f, g) The rates of apoptosis in an H/R model in tubular cells treated with CC with or without pretreatment with irisin versus control groups (*n* = 3 experiments). Statistics are presented as mean ± SEM. ^∗^*P* < 0.05; ^∗∗^*P* < 0.01. Abbreviations: CTL: control, with no addition of irisin; CC: compound C; NS: no significant difference.

To investigate the effect of irisin, the AMPK/UCP2 pathway was assessed in an *in vitro* HK-2 cell model and a mouse model. Firstly, we observed that irisin increased the level of AMPK mRNA (RT-qPCR) and stimulated phosphorylation of AMPK (WB) after renal I/R (Figures 6(a) and 6(b)). As displayed in Figure 6(c), the HK-2 cells treated with irisin exhibited significant activation of AMPK and increased UCP2 expression compared with the cells treated with vehicle. However, pretreatment with CC (AMPK inhibitor) significantly suppressed the irisin effect on mediating the levels of p-AMPK and UCP2 (Figures 6(c)–6(e)). Furthermore, after pretreatment with CC, the effect of irisin in improving cell apoptosis was significantly reduced (Figures 6(f) and 6(g)). These data suggested that irisin increases UCP2 expression through stimulating AMPK phosphorylation.

## 4. Discussion

Numerous studies have shown that irisin is strongly correlated with metabolic diseases [16–18]. However, in recent years, increasing studies have revealed that irisin also participates in the pathophysiological process of various diseases by inhibiting apoptosis, reducing inflammation, and ameliorating oxidative stress [8, 17, 19, 20]. However, whether irisin affects or plays any role in the progression of AKI is unclear. In this study, we chose the classic I/R-induced AKI model and used the intraperitoneal injection of irisin to investigate the role of irisin in AKI progression. Our findings here demonstrated that pretreatment with irisin protected the kidneys of I/R mice against renal tubular epithelial cell damage by increasing UCP2 expression.

Based on previous reports [21, 22], we primarily observed ischemic damage to the proximal tubules in an I/R model. After I/R, biochemical and pathological analyses were conducted to confirm the establishment of the AKI mouse model (Figure 1).

As is well known, oxidative stress and cellular inflammation are involved in the complicated pathophysiology of AKI [22, 23]. Irisin, a 112-amino acid protein released by skeletal muscles after exercise, is thought to have a pivotal role in metabolic disorders. Recently, extensive evidence has suggested that irisin has anti-inflammatory [8, 19, 24], antiapoptotic [25], and antioxidative [8, 17, 20] effects in various pathophysiological processes. Moreover, the correlations between irisin and lung injury [8, 26], cerebral injury [27], and type 2 diabetes [17] have been disclosed, but the effect of irisin in AKI is unknown. Thus, to verify the protective effect of irisin in AKI, we evaluated the levels of various proinflammatory cytokines and oxidative stress in a mouse model. Consistent with previous studies [14, 28, 29], we found that proinflammatory cytokines and oxidative stress were substantially increased after I/R (Figure 2). Nevertheless, as expected, the expressions of TNF-*α*, IL-6, and-1*β*, and MDA were suppressed by irisin. Concomitantly, after injection of exogenous irisin, SOD was significantly higher in the I/R group. Ischemic kidney injury is characterized by the upregulation of adhesion molecules, such as ICAM-1 and P-selectin, to potentiate the interaction of endothelial cells and leukocytes [14, 30]. In our study, we observed the expression of ICAM-1 and P-selectin in kidney tissue by immunohistochemical staining. In accordance with previous studies [30], our results suggested that adhesion molecules and the MPO level were upregulated after I/R injury, indicating endothelial cell activation and leukocyte infiltration. Fortunately, administration of irisin notably ameliorated ICAM-1 and P-selectin staining intensity and the MPO level. Based on the above results, we concluded that irisin exerted renoprotective effects on AKI via inhibiting oxidative stress and cellular inflammation.

In addition, the apoptosis of tubular epithelial cells, a cause of AKI, is responsible for oxidative stress and inflammation [15, 31, 32]. In studies on lipopolysaccharide- (LPS-) and I/R-induced acute lung injury [8, 26] and I/R-caused global cerebral injury [27], the antiapoptotic effect of irisin has been highlighted. In this report, we found pathological changes after I/R accompanied by tubular epithelial cell apoptosis. In support of the pivotal role of irisin in I/R-related apoptosis, we detected apoptosis in the mouse kidneys after 45 minutes of ischemia and 24 hours of reperfusion. In this study, the apoptosis of PTECs after kidney I/R was demonstrated mostly by activation of caspase 3 and the percentage of renal PTEC apoptosis by WB and TUNEL staining, respectively. We observed substantial tubular epithelial cell apoptosis during I/R, and administration of irisin can improve renal injury *in vivo* and *in vitro* through inhibiting apoptosis and decreasing caspase 3 activation in PTECs.

Moreover, in this study, the mice fed with 1% CLA exhibited increased levels of UCP2 and improved renal architecture and renal function. Then, we examined the expression of UCP2 in the kidneys after irisin supplementation by RT-qPCR and found that irisin could increase the level of UCP2 in I/R mice. As a result of upregulating UCP2, renal I/R injury is attenuated by suppressing renal tubular cell apoptosis [7], improving mitochondrial dynamics [6], and decreasing reactive oxygen species (ROS) production [33]. Furthermore, to confirm the potential renoprotective mechanism of irisin in AKI, we used UCP2 siRNA and UCP2 plasmids in the cell model. Fortunately, we observed that cells transfected with p-UCP2 exhibited a significantly reduced H/R-induced cell apoptosis ratio, while H/R cells treated with UCP2 siRNA showed notable cell apoptosis and activation of caspase 3. Concomitantly, the protective role of irisin in the H/R cell model was suppressed, increasing the proportion of apoptotic cells. Based on these data, irisin was confirmed to protect against AKI through upregulating UCP2 expression. In this study, we found that UCP2 was upregulated in kidneys suffering I/R injury, which is in accordance with previous studies [6, 7, 34]. This result may be due to activation of AMPK and accumulation of ROS after I/R injury, which possibly mediates the upregulation of UCP2 expression [34–36]. Interestingly, in a previous report [8], UCP2 was decreased in the lung after I/R damage. This phenomenon may depend on tissue-specific UCP2 expression after I/R-induced injury.

AMPK is a sensor that can respond to changes in energy and metabolism in cells and can be activated by ischemia and hypoxia [37]. In rodents, AMPK is increased and activated in ischemic renal tubular cells and alleviates I/R-induced injury [37, 38]. In this study, we also observed upregulation of AMPK in renal ischemia. Furthermore, our data suggested that AMPK phosphorylation was stimulated by irisin. Therefore, we speculated that AMPK signaling may involve the beneficial effects of irisin on renal I/R. To explore the effect of irisin on AMPK, HK-2 cells were subjected to CC (AMPK inhibitor) pretreatment. As expected, our data implicated that CC reduced the effect of irisin in regulating UCP2 expression and ameliorating cell apoptosis. However, as reported, AMPK activation can upregulate UCP2 [39]. Thus, we speculated that the protective role of irisin on I/R-induced AKI may, at least partly, be associated with activation of the AMPK/UCP2 signaling pathway.

**Figure 7:**
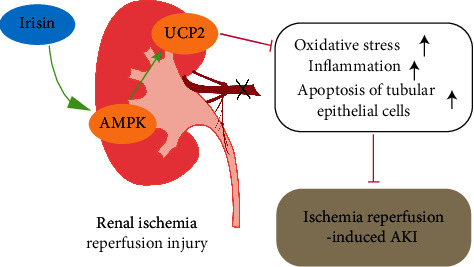
Proposed potential working model of irisin modulation of UCP2 in renal proximal tubular epithelial cells. Irisin activated the AMPK signaling pathway and upregulated the expression of UCP2, which ameliorated I/R-induced renal injury.

In summary, our data indicated that irisin plays a vital role in alleviating I/R-induced AKI by upregulating UCP2 expression, further limiting cellular inflammation and apoptosis. In addition, this study sheds light on the possible role of the AMPK signaling pathway in the renoprotective effects of irisin against I/R-induced AKI (Figure 7). Furthermore, this study mainly focused on the effect of irisin on UCP2 in renal I/R, and further experiments and studies are needed to verify the underlying mechanisms and our data.

## Figures and Tables

**Table 1:** Primer sequences for real-time PCR.

Gene	Forward primer (5′ to 3′)	Reverse primer (5′ to 3′)
AMPK	GGGCACCTGTGGTGCTACCTG	ATGAGCTTTGCCTCCGTCCGC
UCP2	CACCTTCGGCAAAGTGAAGA	TCTTCAACCCTCCCGTGTTT
*β*-Actin	CGTGAAAAGATGACCCAGATCA	TGGTACGACCAGAGGCATACAG

## Data Availability

All data in this study are available from the corresponding authors upon request.
